# Study on the correlation between multimodal image features and multi‐cognitive domain function in patients with cognitive impairment of Qinghai plateau area

**DOI:** 10.1002/alz70856_101815

**Published:** 2025-12-25

**Authors:** Baogen Ru, Aiqin Zhu

**Affiliations:** ^1^ Department of Geriatrics, Qinghai People's Hospital, Xining, China; ^2^ Graduate School of Qinghai University, Xining, China; ^3^ Qinghai Provincial Hospital, Qinghai University, Xining, China; ^4^ Clinical Research Center for Geriatric Diseases of Qinghai, Xining, China

## Abstract

**Background:**

Explore the imaging characteristics of brain structure and function in patients with mild cognitive impairment(MCI) and Alzheimer's disease(AD) in a high‐altitude hypoxic environment, as well as their correlation with multiple cognitive domain.

**Method:**

Based on the Montreal Cognitive Assessment (MoCA) Scales, 30 elderly individuals each from the Qinghai region (average altitude: 2300 meters) with normal cognition (NC), MCI and AD were selected. There were no significant statistically differences among the three groups in terms of age, gender, and education. Multi‐cognitive domain scales were assessed. All participants underwent structural magnetic resonance imaging (sMRI) examinations. Voxel‐based morphometry (VBM) and surface‐based morphometry (SBM) methods were used. Resting‐state functional imaging techniques were analyzed brain network functional connectivity patterns.

**Result:**

Compared with NC group, the volume of gray matter in left fusiform gyrus and right parahippocampal gyrus decreased in MCI group, and the volume and thickness of gray matter in bilateral entorhinal cortex and temporal pole decreased significantly in AD group (*p* <0.05). orrelation analysis revealed that the gray matter volume of left fusiform gyrus was positively correlated with MoCA (r_s_=0.365), Auditory Verbal Learning Test(AVLT‐Delay) (r_s_=0.302) and Verbal Fluency Test (VFT)(r_s_=0.318); The gray matter volume of the right parahippocampal gyrus was positively correlated with MoCA (r_s_=0.355), AVLT‐Delay (r_s_=0.312) and Clock Drawing Test (CDT) (r_s_=0.363) (*p* <0.01). The thickness of the right temporal pole cortex is positively correlated with MoCA scores (r_s_=0.363), AVLT‐delay scores (r_s_=0.318), VFT scores (r_s_=0.310), and CDT scores (r_s_=0.335) (*p* <0.01). Moreover, compared to the NC group, the MCI and AD groups showed significant changes in ALFF, fALFF, and ReHo values in multiple brain regions (*p* <0.05, GRF corrected). The sensorimotor network, ventral attention network and default mode network functional connection were significantly decreased in AD group, and the ventral attention network functional connection was significantly correlated with MoCA (r_s_=0.276) and CDT (r_s_=0.234)(*p* <0.05), although the strength of the correlation was weak.

**Conclusion:**

During the progression from MCI to AD patients in hypoxic environments, there is a gradual deterioration in brain structure and functional connectivity, and these changes are closely related to the decline of overall cognition, episodic memory, language ability and visuospatial function.

